# *NPM1*-Mutated AML: Deciphering the Molecular and Clinical Puzzle in the Era of Novel Treatment Strategies

**DOI:** 10.3390/cancers17132095

**Published:** 2025-06-23

**Authors:** Michael D. Diamantidis, Maria Smaragdi Vlachou, Anastasia Katsikavela, Smaragdi Kalomoiri, Vasiliki Bartzi, Georgia Ikonomou

**Affiliations:** 1 Thalassemia and Sickle Cell Disease Unit, Department of Hematology, General Hospital of Larissa, 41221 Larissa, Greece; smarovlachou96@gmail.com (M.S.V.); akatsikavela@gmail.com (A.K.); 2 Department of Hematology-Lymphoma, Bone Marrow Transplantation Unit, Evangelismos General Hospital, 10676 Athens, Greece; smaragdi5kal@gmail.com; 3 First Department of Propaedeutic Internal Medicine, National and Kapodistrian University of Athens, Laikon General Hospital, 11527 Athens, Greece; vbartzi@yahoo.com; 4 Thalassemia and Sickle Cell Disease Prevention Unit, General Hospital of Larissa, 41221 Larissa, Greece; g.oikonomou@ghl.gr

**Keywords:** acute myeloid leukemia (AML), nucleophosmin (*NPM1*) gene, driver mutation, leukemogenesis, nuclear–cytoplasmic localization, concomitant mutations, measurable residual disease (MRD), menin inhibitors, ziftomenib, revumenib, exportin 1, knockout and knock-in phenotype, clinical trials, *NPM1*-mutated AML, treatment

## Abstract

The aberrant localization of the mutated *nucleophosmin* (*NPM1*) protein in the cytoplasm is the hallmark of the development of acute myeloid leukemia (AML); the gene, located in the nucleolus, codes for a protein that normally shuttles between the nucleus and the cytoplasm of the normal hematopoietic cells. Patients harboring *NPM1* mutations are diagnosed as having *NPM1*-mutated AMLs, which are types of leukemia with distinct clinical and laboratory characteristics. The essential diagnostics for investigating *NPM1*-mutated AMLs, the interactions with concomitant mutations affecting prognosis and the therapeutic interventions that the treatment of such patients requires are discussed in this review. Novel investigational agents in current clinical trials are also highlighted, along with the roles of exportin 1 (XPO1), menin-KMT2A inhibitors and immunotherapy in *NPM1*-mutated AMLs. This review focuses on critically evaluating the available data and aims to reveal the secrets of *NPM1*-mutated AMLs.

## Simple Summary

Acute myeloid leukemia (AML) is an aggressive, heterogeneous blood malignancy harboring various gene mutations. The nucleophosmin 1 (*NPM1*) gene is a common mutated gene in AML, with almost 30% of AML patients having *NPM1* mutations. Despite our increasing knowledge of the pathogenesis and treatment options of *NPM1-mutated AML*, there are many unanswered questions concerning this subtype of leukemia. This study is a detailed review of what is known about *NPM1-mutated AML.* An overview of its definition, subtypes and classification, along with the mutational spectrum that frequently co-exists with *NPM1* mutation in AML and affects prognosis, is provided. The clinical and laboratory characteristics of *NPM1-mutated AML* are also described, with emphasis on disease biology. All important information concerning measurable residual disease (MRD) in patients with *NPM1-mutated AML* is also highlighted. New drugs under development are also presented, as well as how treatment based on precision medicine might improve outcomes. This review attempts to categorize conflicting study results and provide guidance for the future study and optimal treatment of patients with this type of leukemia.

## 1. *NPM1*-Mutated AML: An Overview of Its Definition, Subtypes and Classification

### 1.1. Background

The link between the *nucleophosmin* (*NPM1*) protein and the initiation of acute myeloid leukemia (AML) has been a long-term scientific project. The mutated *NPM1* gene in the nucleus of the leukemic cell, located on chromosome 5q35, was discovered in 2005 by Falini B et al. [1], following the impressive finding of the aberrant localization of the mutated *NPM1* protein in the cytoplasm of leukemic cells with a specific immunohistochemistry (IHC) stain [1,2]. The *NPM1* gene consists of 12 exons and encodes a multifunctional protein comprising 294 amino acids (35–40 kDa) [3,4,5,6]. Normally, the *NPM1* wild type (*NPM1wt*) is located inside the nucleolus of the hematopoietic cells and encodes a multifunctional protein, which shuttles between the nucleolus, the nucleus and the cytoplasm. The normal functions of *NPM1wt* in the nucleolus include controlling centrosome duplication to ensure effective cell division, regulating ribosome biogenesis, forming heterochromatin to control standard gene expression, ensuring the stability of DNA repair and enhancing *TP53* transcriptional activity, among others. It must be mentioned that most of this functional workload is mediated by its potential to chaperone RNA molecules from the nucleus to the cytoplasm. This nucleocytoplasmic transport is essential for the cellular regulatory and maintenance processes [3,7,8,9]. Hence, the location of the *NPM1* gene (either in mutated or wild-type form) is the nucleus of the hematopoietic cells, whereas the aberrant localization of the *NPM1-mutated* protein, which can be identified via IHC staining on a bone marrow (BM) biopsy, occurs in the cytoplasm. IHC was the initial method used for identifying the *NPM1-mutated* protein, long before the advent of next-generation sequencing (NGS) data [10,11].

### 1.2. NPM1 Mutational Landscape

As highlighted, the hallmark of *NPM1-mutated* AML is the cytoplasmic localization of the mutant protein, primarily due to specific genetic alterations, as opposed to the natural nucleolar habitation of the *NPM1*-mutated and *NPM1wt* genes. The latter is attributed to the *NPM1wt* C terminus amino acid positions W288 and W290, which are highly conserved and indispensable for forming a globular structure, responsible for the nucleolar localization of the gene [7]. Conversely, *NPM1* mutations delete or alter key amino acids. Thus, the C terminus of the *mutated NPM1* loses the amino acid tryptophan in positions W288 and W290 (or W290 alone), causing the disruption of the folded helix structure, the loss of the nucleolar localization signal and the aberrant cytoplasmic localization of *NPM1* mutants, as observed in AML [7,11,12,13,14]. As a result of these mutations, the mutant *NPM1* protein loses its nuclear localization signal. The latter acquired characteristic leads to the cytoplasmic export and retention of the *NPM1-mutated* protein, a defining hallmark of *NPM1-mutated* AML. Despite its high prevalence, the mechanisms of action of the cytoplasmic *mutated NPM1* protein in AML remain poorly understood [4,12].

Normally, nuclear import predominates over export, so the normal *NPM1* protein mainly remains in the nucleus. However, when *NPM1* mutants are present, nuclear export predominates over import and the *NPM1* proteinic mutants are relocated in the cytoplasm. In other words, *NPM1* mutants are ‘born to be exported’ [7,8,13].

*NPM1* mutations are categorized based on variations in their mutational spectrum. Type A mutations harbor the combination of TCTG between nucleotides 860 and 863 and type B mutations insert CATG between nucleotides 863 and 864, whereas type D mutations add CCTG between nucleotides 863 and 864 [3,15]. Almost 70% of the total *NPM1* mutations are type A, while 11% are type B. Type A mutations are very often observed with concomitant *DNMT3A* mutations, which are responsible for worse prognoses in type A and type D *NPM1-mutated* AML [15]. Intriguingly, the mutated *NPM1* allele exerts a dominant biological behavior over the wild-type *NPM1* allele due to the preferential transcription of the mutated *NPM1* allele [16]. As a result, the mutant/normal *NPM1* heterodimers are relocated to the cytoplasm of the leukemic cells [17].

*NPM1* mutations in AML are further classified based on the number of the lost tryptophan residues: the mutations causing the loss of two tryptophan residues are characterized as A-like, in contrast to those inducing the loss of one tryptophan residue, which are characterized as non-A-like, more extensively studied in pediatric AML [18]. Patients with non-A-like *NPM1-mutated* AML have a better prognosis and increased sensitivity to chemotherapy, probably because *NPM1* partially remains in the nucleolus. Contrarily, patients with A-like *NPM1-mutated* AML have a more dismal prognosis and a disease with a more aggressive clinical course. They exhibit a decreased response to chemotherapy, mainly because of HOXA5, HOXA10 and HOXB5 up-regulation and p14ARF/p21/p53 pathway deregulation [18].

### 1.3. Geographic and Ethnic Variations in NPM1 Mutation Prevalence

Several studies have yielded geographical and ethnic differences in the prevalence of *NPM1* mutations among AML patients. These differences are particularly age-dependent and vary significantly between Western and Asian populations. For example, a higher prevalence of this mutation in older AML patients has been observed in Europe compared with Asian countries, such as China, Japan and Korea [19,20,21,22,23]. This mutation is quite common in newly diagnosed (ND) AML patients: it is found in around 30% of cases and its prevalence shows minor variations across different European registries, ranging from 27% [24] to 33% [25]. However, Asian AML patients show lower percentages of the *NPM1* mutation, ranging from 13.3% to 28%. The latter could indicate a potential ethnic effect on the probability of *NPM1* prevalence [20,21,22,23].

The observed age variations in the mutational landscape between younger and older adults in AML [26] do not influence the aforementioned ethnic differences. Thus, *NPM1* occurs in 23.6% of patients older than 40 years old in the Chinese data, significantly lower than in the German AML2003 data, even after age adjustment [20]. Interestingly, lower frequencies of *NPM1* mutations have also been reported in Black AML patients [27]. Indeed, Black ethnicity is an independent prognosticator of poor survival in AML patients, probably because fewer *NPM1* and more *IDH2* mutations are observed in younger Black patients [28]. Intriguingly, the OS of younger Black AML patients in contrast to White patients was not improved by *NPM1* mutations, even though this measure was adversely affected by *FLT3-ITD* and *IDH2* mutations [28].

The reasons for these genetic discrepancies among international cohorts and the biology of the disease behind these differences are largely unknown. It could be hypothesized that environmental factors affect the epigenetic modification of the genome, whereas the complex interplay between biological (genetic predisposition) and environmental factors causes genetic variations. Due to the disease heterogeneity, particular AML subtypes may be biologically distinct across different ethnic backgrounds, potentially affecting mutation rates. All these ethnic differences suggest a need for different treatment intensities and modalities in specific AML subgroups, which will be the intended outcomes of well-designed clinical trials comparing different ethnic populations. The latter might lead to different prognostic risk grouping and variations in AML treatment in different parts of the world.

### 1.4. Classification of NPM1-Mutated AML

In every recent AML classification system, ranging from the one comprising 13 subgroups proposed by Papaemmanuil E et al. in 2016 [24] to the newest involving 16 distinct subgroups suggested by Tazi Y et al. in 2022 [29], AML with the *NPM1* mutation is classified as a separate category. The International Consensus Classification (ICC) of 2022 classifies AML with mutated *NPM1* as a separate category requiring 10% blasts or more in peripheral blood (PB) or BM for establishing an AML diagnosis [30]. Likewise, the *NPM1* mutation is considered to be an AML-defining genetic abnormality, also forming the entity of AML with mutated *NPM1* in the European Leukemia Net (ELN) 2022 Guidelines [31]. This classification has also incorporated *NPM1* mutations as AML-differentiating genetic lesions, thereby confirming these mutations’ unique position within the subtypes of AML [31]. Similarly, in the 5th World Health Organization (WHO) 2022 classification, AML with *NPM1* mutations belongs to the diagnostic group known as AML with defining genetic abnormalities [32]. The only important difference compared to the ICC 2022 is that in the WHO 2022 classification, no blast enumeration threshold exists as a prerequisite for diagnosis, as AML with *NPM1* mutation is diagnosed irrespective of the blast count [32,33,34].

### 1.5. NPM1 as a Genetic Driver Mutation in AML Initiation: The Never-Alone, Usual Suspect

The nature of a ‘driver’ mutation must be justified based on its ability to independently initiate a neoplasm without requiring additional genetic alterations. One way to test whether a mutation meets this criterion is by replicating it in a mouse model and observing whether leukemia develops.

It was reported that the activation of a humanized *NPM1* cytoplasmic knock-in allele in mouse hematopoietic stem cells caused delayed-onset AML in one third of the mice, along with HOX gene overexpression, suggesting that additional mutations are needed in this model to initiate leukemogenesis [35]. To determine which additional mutations could accelerate this process, scientists used a transposon system that randomly inserts itself into the genome, disrupting various genes. In 2011, these results provided initial insights showing that mutant *NPM1*, in co-operation with other secondary mutations, drives leukemia initiation and progression in mice. Furthermore, possible therapeutic targets, such as HOX, were identified in *NPM1*-mutated AML [35].

**Figure 1:**
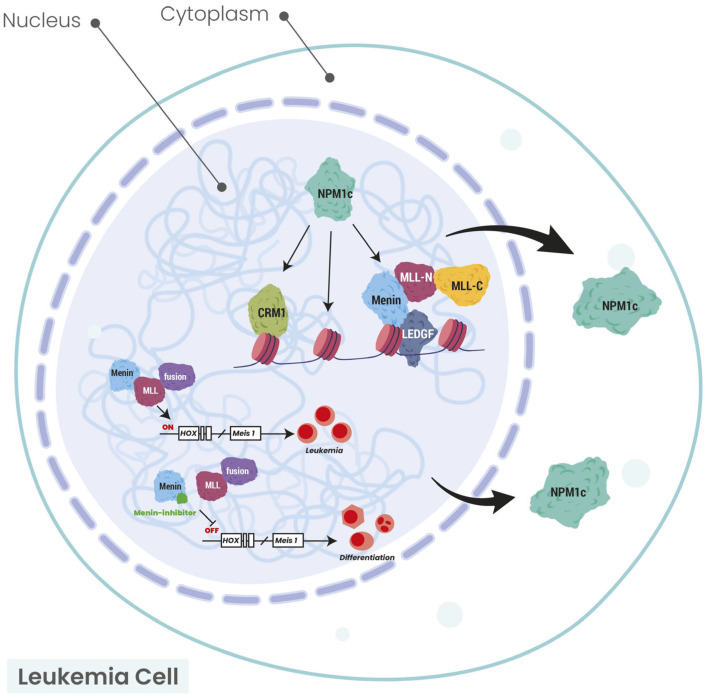
Schematic illustration of the connection between *NPM1* and HOX/MEIS1/XPO1/menin axis. The arrows show the cytoplasmic localization of NPM1 protein in *NPM1*-mutated AML. Modified from Uckelmann HJ et al. [37] and Krivtsov AV et al. [38]. CRM1 chromosomal region maintenance 1 (XPO1: exportin 1); HOX: homeobox; LEDGF: lens epithelium-derived growth factor; MEIS1: MEIS homeobox 1; MLL: mixed lineage leukemia; MLL-N: mixed lineage leukemia—nuclear; MLL-C: mixed lineage leukemia—cytoplasmic; NPM1: nucleophosmin 1; NPM1c: nucleophosmin 1 cytoplasmic.

Recently, a well-designed study showed that *NPM1* haploinsufficiency in collaboration with MEIS1 overexpression is sufficient to induce complete AML that transcriptionally resembles human *NPM1-mutated* AML in mice [36]. Moreover, the MEIS1-SMC4 axis is a potential therapeutic target in this type of AML. The latter experiment showed that *NPM1* mutations, in collaboration with DNA-binding transcription activators, such as MEIS1, can drive AML. Until today, the downstream targets or effectors of the *NPM1*-mutated protein, such as HOX, exportin-1 (XPO1) or the menin-KMT2A axis, have been inhibited in clinical trials. These treatment approaches are highlighted in the section of this manuscript describing the treatment of *NPM1*-mutated AML. All the above notions are demonstrated in Figure 1.

As more NGS data became available, it became evident that *NPM1* mutations rarely occur in isolation, instead co-existing with other genetic alterations, reinforcing the idea that they function within a broader leukemogenic framework. However, *NPM1* mutation is considered to be a driver mutation and a major gene causing leukemogenesis by the scientific community, probably due to its leading role and tight connection with AML pathogenesis [3,4,7,12], even though it does not completely fulfill the strict definition of initiating AML alone in the mouse models, having a strong influence on and collaborating with MEIS1 overexpression to induce leukemia [36].

### 1.6. The Role of Genomic Instability and Additional Mutations in AML Development

Genomic instability, defined as an increased tendency of the genome to acquire mutations, is a common mechanism in cancer. Nevertheless, in AML, genomic instability is uncommon. Most of the mutations in AML are random and occur in hematopoietic stem cells or progenitors before the acquisition of the initiating mutation. Only one or two additional mutations in combination are required to generate the initial neoplastic clone [39].

It is important to state that many genes are recurrently mutated in AML; moreover, individual leukemias harbor multiple mutations (molecular aberrations or defects) and are potentially composed of subclones with different mutational compositions, genetic profiles and leukemogenic capacities, rendering each patient’s AML genetically unique. Furthermore, the biology of AML significantly differs between younger and elderly patients. Driver mutations such as *DNMT3A*, *TET2*, *IDH2*, *TP53*, *RUNX1*, *NPM1*, *NRAS*, *FLT3-ITD*, *FLT3-TKD*, *ASXL1* and *STAG2* are often enriched in elderly AML patients (>65–70 years old), thereby differing in type and prevalence from those encountered in younger patients, who exhibit a comparatively different mutational landscape [19,26,40]. *DNMT3A*, *TET2*, *ASXL1* and other genes are also involved in the *PRC1/PRC2* regulation circuit, offering another point of view on gene regulation in AML.

There are emerging data suggesting that cases previously classified as Myelodysplastic syndromes (MDSs) or MDS/myeloproliferative neoplasms (MPNs) with *NPM1* progress to AML shortly. The latter means that *NPM1* mutation is linked with AML development, as a driver mutation, in a small period from the time of acquisition of the mutation, causing either de novo or secondary AML. Similar findings also exist from patients with clonal hematopoiesis (CH) who acquire an *NPM1* mutation; AML evolution occurs within a short period of time [32]. Thus, *NPM1* mutation is strongly correlated with the presence of AML and the emergence of AML, shortly after the diagnosis of MDS and MDS/MPN. For all the above reasons, the acquisition of an *NPM1* mutation is considered a driver mutation at the clinical level [3,7,12] and a later event in leukemogenesis [24]. *NPM1* mutations act as ‘gatekeepers’ for AML, mainly de novo AML [7]. These mutations are found in the entire leukemic population by IHC; they are stable over time, also being detected at relapse; and they are related to the mutated protein, causing its production. This is why *NPM1* gene transcripts are an excellent marker for evaluating measurable residual disease (MRD) in AML [25,41,42].

### 1.7. NPM1 as an MRD Marker in AML

According to 2022 ELN guidelines, MRD monitoring in patients with *NPM1*-mutated AML is critical in evaluating disease response to drugs, guiding post-remission therapy and, in parallel, predicting relapse [31]. There are established guidelines for monitoring AML patients with *NPM1* mutations under intensive induction chemotherapy regimens, whereas there are no current guidelines for MRD testing for patients receiving non-intensive venetoclax (VEN)-based regimens.

Real-time quantitative PCR (RT-qPCR) is the recommended method for detecting *NPM1* mutations because of its high sensitivity and specificity [31,42]. Thus, MRD should be assessed with *NPM1* transcripts (PCR) in the PB after two cycles of intensive chemotherapy, in the BM at the end of treatment, and either in the BM every 3 months or in the PB every 4–6 weeks for 24 months after treatment completion [31,42,43]. These guidelines were derived from patients with *NPM1*-mutated AML who achieved CR after two cycles of intensive induction chemotherapy.

MRD positivity is defined as ≥2% in the BM or failure to achieve a 3 to 4 log reduction in either the BM or PB at completion of consolidation. Molecular MRD detectable at a low level (MRD-LL) is defined as <2% and designated as negative, because when measured at the end of consolidation treatment, it is associated with a very low relapse rate. MRD relapse is defined as conversion from MRD negativity to MRD positivity or an increase in MRD ≥ 1 log_10_ between any two positive samples for patients with MRD-LL [42,43,44].

Othman et al. evaluated patients with ND *NPM1*-mutated AML regarding their MRD status. These patients achieved CR after therapy with VEN plus HMAs or low-dose cytarabine. MRD was assessed via RT-qPCR. Achieving BM MRD negativity after the end of four cycles of therapy was linked with the greatest improvement in OS; in particular, it predicted a better 2-year OS (58% exhibited BM MRD negativity) [45]. Several issues arose because of these important results. It is not clear if MRD-negative patients at the end of cycle 4 should continue treatment with VEN plus HMAs, decrease the dosages or the days exposed to the administered drugs or even stop therapy [46]. The latter is extremely important, as long exposure to VEN might lead to resistance to the drug due to the appearance of novel *KRAS* or *NRAS* mutations [47]. Interestingly, for MRD-positive patients at the end of cycle 4, preemptive therapy is proposed by some clinicians, with significant advantages and disadvantages. Moreover, other issues regarding MRD in AML patients who received VEN/HMAs include the role of MRD in earlier cycles, defining the most clinically actionable MRD level and selecting either the BM or the PB as a better strategy for MRD monitoring [46].

### 1.8. The Order of Mutations in AML Development

It has long been known that the primary leukemogenic mutations are usually epigenetic modifiers (*TET2*, *DNMT3A*, *IDH1/2*, *ASXL1*, because they are part of the *PRC1*/*PRC2* circuit), followed by *NPM1* or *RAS* mutations that eventually lead to AML progression [24]. Thus, the AML mutations that can be found together or at different time intervals and the reasons why this occurs are known in individual AML patients. For example, *DNMT3A* mutations and type A *NPM1* mutations co-operate to initiate leukemogenesis. Further molecular lesions in *FLT3-ITD* drive proliferation, setting the stage for expanding and establishing a predominant clone [3,7]. The order of the mutations in time can be indirectly inferred by the Varied Allele Frequency (VAF), defined as the number of alleles harboring the respective mutation compared to the normal alleles. A higher VAF is indicative of an earlier mutation in the complex mutational leukemogenic landscape in an individual AML patient [24]. These insights highlight the sequential nature of AML progression, where certain mutations establish a foundation for leukemogenesis, whereas others contribute to disease aggressiveness.

### 1.9. The Functional Impact of NPM1 Mutations on Leukemia Biology

At the molecular level, the *NPM1* mutant protein interacts with other oncogenic proteins, engaging with various oncogenic pathways within the leukemic cell cytoplasm. A significant interaction occurs with FOXM1, a transcription factor trapped in the cytoplasm by mutated *NPM1,* hindering its normal transcriptional function. *NPM1* mutations are also associated with increased up-regulation of the MLL/MEIS1/HOX axis, a critical effect for maintaining the leukemic stem cell fate [48]. Another oncogenic mechanism of the *NPM1* mutation involves the nuclear export of the tumor suppressor p14 (ARF), causing a poor p53 response, with the cytoplasmic export of PU.1 resulting in the suppression of more than 300 myeloid differentiation genes or the overexpression of the oncogenic long-noncoding RNA LONA, which becomes nuclear as the mutant *NPM1* protein relocalizes into the cytoplasm [48].

Additionally, while *NPM*, *FLT3-ITD* and *DNMT3A* mutations rarely co-occur in AML, they interact to drive the increased expression of the transcription factor hepatic leukemia factor (HLF) [49]. The result is an adverse phenotype with an increased leukemic stem cell burden with an aberrant immunophenotype [CD34-low, G protein-coupled receptor 56 (GPR56)-high] harboring a poor prognosis [49]. Furthermore, the aforementioned leukemic phenotype is significantly related to HOX and histone H1 signal transduction pathways, associated with transcriptional misregulation and clinically with older age, lower complete remission (CR) rates and poor clinical outcome [50].

## 2. Clinical and Laboratory Characteristics of *NPM1*-Mutated AML

### 2.1. Chromosomal Aberrations of NPM1-Mutated AML

Most AML patients harboring *NPM1* mutations have normal karyotypes (85% of total patients). The remaining 15% of patients carry chromosomal aberrations, the most frequent being +8 (trisomy), del(9q), +4 (trisomy), −Y, +21 (trisomy) and monosomies 5 and 7 [7,14,51,52]. These aberrations are considered secondary late events during the clonal evolution of *NPM1*-mutated AML [11,52]. Such leukemias show overlapping biologic, pathologic and immunophenotypic features. Prognosis is variable in all the above-mentioned abnormalities, also depending on the presence of other mutations, along with the *NPM1* mutation and the cytogenetic lesions [53,54,55,56]. Intriguingly, all AML patients with del(9q) as a sole abnormality consistently display a distinct, exclusive combination of *DNMT3A* and *NPM1* mutations [56].

It is well established that *NPM1-ALK* fusions are very common in anaplastic large-cell lymphoma (ALCL) patients [57]. ALCL is a type of T-cell non-Hodgkin lymphoma (NHL). A few cases harboring *NPM1-ALK* fusions have been documented in B-cell acute lymphoblastic leukemia (ALL) [58]. Interestingly, oncogenic *ALK* point mutations (A348D and F856S) have also been discovered in a B-ALL and an AML patient, respectively [59]. *ALK* fusions have very rarely been observed in AML patients [60,61].

### 2.2. Demographics, Clinical Presentation and Immunophenotypic Features

*NPM1*-mutated AMLs show female predominance, with increased White Blood Cells (WBCs) and platelets and a lower incidence in children (2–8%) [17,51,62]. Moreover, there is a frequent association with extramedullary involvement, particularly in the skin, which is easily diagnosed via IHC. The bone marrow is highly hypercellular, with high blast percentages in most cases, and cup-like nuclei are observed [63], whereas multilineage dysplasia is evident in almost 20% of patients [51]. Even though all FAB categories can be represented, myelomonocytic (M4) and monocytic (M5) types are the most often encountered [10,17,62,64]. The high mutation incidence of *NPM1* (38%) may partly explain the relatively benign clinical manifestations of FAB M4 and M5 AML [65]. The majority of these patients have favorable prognosis [31] and do not undergo hematopoietic stem cell transplantation (HSCT). However, a recent study highlighted that the combination of *NPM1* and *FLT3-ITD* mutations (intermediate prognosis) correlated with a significantly higher incidence of M5 morphology (67.4%) compared to *NPM1*-positive and *FLT3-ITD*-negative mutations (54.5%) [66]. Thus, due to the known limitations of the retrospective nature, the small sample size and the lack of evaluation of co-existing mutations of the conducted study [65], larger prospective trials incorporating different age subgroups and various applied treatments, along with the presence of concomitant mutations, are necessary to assess the effects of possible allogeneic HSCT in *NPM1*-positive, FAB M4 and M5 AML patients.

The number of WBCs in *NPM1*-mutated AMLs increases progressively from concomitant *FLT3-ITD* wild-type to *FLT3-ITD* mutated cases, being much higher comparatively in the latter type. There is no or low expression of CD34, while CD34 positivity has been linked with adverse outcomes; on the contrary, the blastic cells of *NPM1*-mutated AMLs exhibit increased CD33 expression [67]. The response of *NPM1*-mutated AMLs to the induction of chemotherapy is excellent [10]. It is important to emphasize that *NPM1* mutants are restricted to myeloid cells and are never found either in B or T cells in BM or PB [68].

### 2.3. Diagnostic Challenges of NPM1-Mutated AML

Another important element diagnostically is that even though the majority of cases harbor *NPM1* mutations in exon 12 (99%) [10], there are extremely rare cases (<1%) involving exons 11 [69], 5 [70] and 9 [71]. Diagnosing such cases can be challenging, as these very rare mutations may be missed. That is why IHC should always be conducted in BM biopsies in AML patients for detecting the cytoplasmic expression of *NPM1*, which is a surrogate for *NPM1* mutations [1]. Thus, when there is discrepancy between a positive IHC (cytoplasmic *NPM1*) and a negative conventional molecular analysis of exon 12 (absence of exon 12 *NPM1* mutation), there is an *NPM1* mutation in another exon, suggesting that the entire coding sequence of *NPM1* should be investigated by properly modifying the NGS panels, as the commercially available NGS panels are designed to recognize only *NPM1* mutations of exon 12 [11]. Thus, such patients are properly classified prognostically as belonging to the ELN-favorable risk group (mutated *NPM1* in exon 11 without *FLT3-ITD*), instead of being wrongly classified in the ELN-intermediate risk group (*NPM1* wild-type without *FLT3-ITD*) [31]. MRD monitoring in these rare *NPM1*-mutated AMLs requires designing a patient-specific real-time q-polymerase chain reaction (RT-qPCR) assay [41].

Intriguingly, the principle that *NPM1* gene transcripts are an excellent marker for evaluating MRD in AML has some even more extreme rare exceptions. Indeed, *NPM1*-mutated, fit, older AML patients (≥65 years) at diagnosis have been reported to relapse and acquire resistance with negative *NPM1*-mutated transcripts after the application of VEN plus modified intensive chemotherapy (CAVEAT study) [72,73]. In these AML patients, the *NPM1* gene could not be used as a marker for evaluating MRD status, since it was negative at relapse and not positive, despite initial positivity at diagnosis [72]. The anti-apoptotic proteins MCL-1 and BCL-XL exhibited an elevated expression at the relapse of the *NPM1-mutated* AML, and this up-regulation was established via flow cytometry [72]. In contrast to younger populations, *NPM1-mutated* AML confers a favorable prognosis in older AML patients ≥ 65 years old, who experience durable treatment-free remission with the above-mentioned time-limited regimen (CAVEAT study) [73].

### 2.4. Essential Diagnostics for Investigating and Treating NPM1-Mutated AML

The tests and procedures used at diagnosis for a patient with AML were proposed by the ELN 2022 recommendations [31]. Beyond the routine tests to establish the diagnosis and necessary cytogenetics within 5–7 days, the following screening process for gene mutations required for diagnosis, risk stratification and the identification of actionable therapeutic targets is necessary: *FLT3-ITD*, *FLT3-TKD*, *IDH1*, *IDH2*, *NPM1* (within 3–5 days), *CEBPA*, *DDX41*, *TP53*, *ASXL1*, *BCOR*, *EZH2*, *RUNX1*, *SF3B1*, *SRSF2*, *STAG2*, *U2AF1*, and *ZRSR2* (within the first treatment cycle). The screening process for routine gene rearrangements should also be performed within 3–5 days. The following additional genes are recommended to be tested for at diagnosis: *ANKRD26*, *BCORL1*, *BRAF*, *CBL*, *CSF3R*, *DNMT3A*, *ETV6*, *GATA2*, *JAK2*, *KIT*, *KRAS*, *NRAS*, *NF1*, *PHF6*, *PPM1D*, *PTPN11*, *RAD21*, *SETBP1*, *TET2*, and *WT1* [31]. This is also significant, as mutations co-existing with an *NPM1* mutation affect prognosis and alter the risk stratification of the patient (analyzed in the risk stratification part of this manuscript). In cases of delayed screening results not only for *NPM1* but also for the other above-mentioned key mutations, important information regarding the prognostic classification of the AML patient is lacking at the critical time point for making a treatment decision on whether to transplant the patient or not.

Moreover, biobanking and the proper registry of family medical history are extremely important for both ongoing research and future therapeutic developments. Advances in genetic testing, in combination with a detailed family history, have enabled identifying germline predisposition syndromes [74]. The HSCT donors carrying deleterious germline variants, such as *RUNX1* and/or *CEBPA*, should be excluded from transplantation [31]. Furthermore, AML patients harboring a germline mutation should be under close surveillance concerning their donor selection, optimal timing for HSCT, conditioning regimen, and comorbidities, aiming to minimize toxicities [74]. Ensuring the proper biobanking of biologic tissue is of the utmost importance for enhancing research and therapeutic strategies. Recent studies have investigated the predisposition potential of several germline variants in large cohorts of AML patients [75] or evaluated the clonal evolution and early detection of *DDX41*-mutant AML by defining the risk associated with *DDX41* variants [76]. The latter method enables closer AML monitoring and disease management for patients and their families.

## 3. The Prognostic Complexity of *NPM1*-Mutated AML

*NPM1*-mutated AML is considered a separate entity in most classifications. However, it is contradictory and peculiar that this mutation is never observed alone, but always with other mutations, affecting and modifying the clinical course of the individual AML patient. It is, therefore, essential to note that *NPM1*-mutated AML should always be evaluated in the constellation of mutations in the specific individual with AML. In addition, such an evaluation requires NGS data for proper risk assignment.

Large patient studies have revealed the mutations most frequently co-existing with *NPM1* mutations. In a cohort of 418 patients harboring *NPM1* mutations, the most common co-mutated genes were as follows: *DNMT3A* (54%), *FLT3-ITD* (39%), *NRAS* (19%), *TET2* (16%) and *PTPN11* (15%) [24]. Almost identical results for *DNMTRA*, *FLT3-ITD* and *PTPN11* with the addition of *cohesin Complex mutations* (20%), *IDH1* (15%) and *IDH2^R140^* (15%) were reported in ND AML patients [25]. A more recent NGS analysis of 71 patients with NPM1-mutated AML yielded the following results: *DNMT3A* (62%), *FLT3-ITD* (38%), *FLT3-TKD* (14%), *TET2* (27%), *IDH2* (23%), *IDH1* (18%), *PTPN11* (18%) and *NRAS* (17%) [77].

Interestingly, the combinations of *NPM1* mutations with *IDH2^R172^* for all AML patients and the mutational combinations *NPM1/TP53*, *NPM1/RUNX1* and *NPM1/ASXL1* for elderly patients are mutually exclusive [19]. The data highlight that an *IDH2* mutation usually remains in CR and precedes an *NPM1* mutation in leukemogenesis, whereas an *IDH1* mutation does not remain in complete remission (CR) and is usually subclonal [77]. The remains of an *IDH2* mutation and the hierarchy of the *IDH* clone in CR with the concomitant clearance of an *NPM1* mutation do not affect overall survival (OS) [77].

Regarding prognosis, the presence of other molecular defects, along with *NPM1*, influence the outcome of the individual AML patient. In general, AML patients with an *NPM1* mutation in the absence of *FLT3-ITD* belong to the favorable prognostic risk category (ELN 2022 risk stratification for patients receiving intensive treatments) [31]. These fit patients are treated with chemotherapy without HSCT, while the unfit patients or those >75 years old are treated with a combination of VEN plus a hypomethylating agent (VEN-HMA). However, when the *NPM1* mutation co-exists with the *FLT3-ITD* mutation [29,31], the prognostic risk category is intermediate, and HSCT after chemotherapy is necessary for fit patients. Moreover, patients with the *NPM1* mutation and adverse-risk cytogenetic abnormalities, even in the absence of *FLT3-ITD* mutation, are categorized as adverse-risk patients [31], indicating the inadequate tumor suppressor potential of the *NPM1* mutation and the predominance of cytogenetic risk over molecular risk [14].

For patients receiving less intensive treatments, an important factor affecting the prognosis and the response to VEN-azacitidine (AZA) in *NPM1-mutated AML* is the co-presence of activating signaling mutations (*FLT3-ITD*, *KRAS*, *NRAS*). *NPM1*-mutated AML when these mutations are absent has a median OS of 39 months; in contrast, this figure decreases to 9.9 months when these kinase-signaling mutations are present in parallel with the *NPM1* mutation [78,79].

Moreover, for patients receiving less intensive treatments, *TP53*, *KRAS*, *NRAS* and *FLT3-ITD* guide prognosis and classify AML patients who received VEN-AZA into three different prognostic groups. A favorable-risk group with all involves four genes being negative (median OS: 26.5 months), an intermediate-risk group has positive FLT3-ITD, mutated KRAS and/or mutated NRAS (median OS: 12.1 months) and an adverse-risk group has mutated TP53 (median OS: 5.5 months) (2024 ELN recommendations) [78]. Thus, a patient with *NPM1*-mutated AML who received VEN-AZA will have a favorable prognosis if *TP53*, *KRAS*, *NRAS* and *FLT3-ITD* are negative, while their prognosis will be intermediate in case of positivity of *FLT3-ITD*, *KRAS* and/or *NRAS* (Table 1) [78,79,80].

NGS data play a crucial role in stratifying AML risk. Although the clinical application of these data can sometimes be contentious, they are instrumental in identifying prognostically significant mutations. As an example, AML patients with an *NPM1* mutation and no *WT1* mutation are stratified as favorable-risk individuals, whereas the concomitant presence of both mutations significantly affects prognosis, so such patients are categorized as adverse-risk individuals [88]. Patients stratified as adverse-risk have a reduced survival rate, and HSCT is the optimal treatment for those who are eligible.

In general, the combination of mutations in *NPM1* and *IDH* (either *IDH1* or *IDH2*) is considered a favorable profile, especially for those AML patients destined to be treated with VEN plus HMA [77,89]. A better OS after CR has been observed for patients harboring both mutations compared to mutated *NPM1* with wild-type *IDH1-2*. Intriguingly, *R140 IDH2* mutations are highly associated with *NPM1* mutations [90]. The association of *NPM1* with *R140 IDH2* might justify the favorable prognosis of *R140 IDH2*-mutated AML, especially in patients receiving VEN-AZA [91,92]. Furthermore, the triple combination of mutated *NPM1*/*DNMT3A*/*NRAS^G12/13^* has been linked with a favorable prognosis, whereas mutated *NPM1*/*DNMT3A*/*FLT3-ITD* has been associated with an intermediate [77] or adverse [49,50] prognosis, as expected from the presence of *FLT3-ITD*.

Interestingly, genomic and clinical data from 1961 AML patients analyzed via a machine learning process have been categorized as follows: combinations of mutated *NPM1*/wild-type *IDH1-2*, mutated *NPM1*/mutated *IDH2*, mutated *NPM1*/wild-type cohesin/mutated *NRAS* and mutated *NPM1*/*mutated* cohesin/mutated *FLT3-ITD* were categorized as good risk with a 60–80% 4-year OS, while the combination of mutated *NPM1*/mutated *IDH1* was classified as intermediate risk with a 40–60% 4-year OS [93]. The triple combination of mutated *NPM1*/*mutated* cohesin/mutated *NRAS* is extremely favorable, with a more than 80% 4-year OS, while the combination of mutated *NPM1*/*DNMT3A*/*FLT3-ITD* is classified as poor risk (20–40% 4-year OS) [93].

Finally, the prognosis of AML when *NPM1* mutations (in the absence of *FLT3-ITD* mutations) co-exist with myelodysplasia-related gene (*MRG*) mutations (*ASXL1*, *BCOR*, *EZH2*, *RUNX1*, *SF3B1*, *SRSF2*, *STAG2*, *U2AF1* or *ZRSR2*) remains controversial. One recent German study found that these secondary-type mutations do not impact the favorable outcome of *NPM1-mutated* AML (no HSCT) [94,95], whereas another synchronous American study showed that AML patients with co-mutations in *MRGs* and *NPM1* harbor a survival similar rate to ELN 2022 intermediate risk when treated with intensive chemotherapy without VEN and that, thus, fit patients should undergo HSCT [96].

Other large studies have also confirmed that the presence of *MRG* and *NPM1* mutations, in the absence of *FLT3-ITD*, does not alter the favorable prognoses and treatment outcomes of patients with *NPM1*-mutated AML [97,98]. Intriguingly, Wang Y et al. found a statistically significant worse progression-free survival (PFS) and non-OS for AML patients with *NPM1* and *MRG* mutations, even though patients with concomitant *FLT3-ITD* mutations exhibited OS and PFS comparable to those with MRGs [97]. Zhao D et al. also demonstrated that *NPM1*-mutated AML, after a previous diagnosis of MDS or MDS/MPN, is enriched in MRGs and has an inferior prognosis compared to de novo *NPM1*-mutated AML [99].

However, there are studies highlighting that AML patients with *MRG* and *NPM1* mutations should be classified as intermediate-risk individuals, not as favorable-risk individuals, due to the negative impact of MRGs in the prognosis of *NPM1*-mutated patients [100]. In a recent series of 568 patients with *NPM1*-mutated AML treated with intensive chemotherapy, those harboring MRGs had significantly decreased event-free survival (EFS) and a higher probability of MRD-positive disease at the end of induction [101].

More precisely, age, *DNMT3A* (*R882 variant*), *IDH1* and MRGs have been identified as unfavorable factors for EFS, while mutations in the cohesin complex and treatment with gemtuzumab ozogamicin (GO) have been described as favorable EFS factors in *NPM1*-mutated AML [101]. The inferior EFS associated with MRGs in *NPM1*-mutated patients does not remain when data from *NPM1-mutated* MRD analysis post-cycle 2 are included. Nevertheless, *DNMT3A* (*R882* variant) and *MYC* mutations retain their adverse effect and cohesin complex mutations have a favorable effect on EFS, regardless of the *NPM1*-mutated MRD status [101].

The reasons for these discrepancies are unknown. A different selection of the population of patients, a different type of *MRG* mutation for each patient or a varied order of acquisition of these mutations might explain the conflicting results of these studies, which were derived at recognized institutions.

## 4. Targeted Agents

### 4.1. NPM1-Mutated AML and Exportin 1 (XPO1)

XPO1 is a nuclear transporter, acting as a direct carrier of *NPM1* mutants from the nucleus to the cytoplasm, favoring leukemogenesis [102]. The latter causes the aberrant cytoplasmic localization of *NPM1* proteins and in parallel promotes the high expression of homeobox (HOX) genes [103,104], along with the relevant co-factors MEIS1 and PBX3 [105,106,107], critical for oncogenesis [103]. Thus, XPO1 inhibition might serve as a promising targeted strategy for *NPM1*-mutated AMLs [103]. Novel drugs, such as selinexor or eltanexor, known as selective inhibitors of nuclear export (SINEs), are under evaluation in several clinical trials for AML patients.

Selinexor (KPT-330) administered once or twice weekly in a phase I clinical trial (NCT01607892) did not show significant clinical efficacy in *NPM1*-mutated AMLs. On the contrary, grade ≥ 3 toxicities, such as neutropenia, thrombocytopenia, fatigue and anorexia, were observed [108]. The above-mentioned dosing strategy for selinexor, resulting in a temporary disruption of the XPO1-*NPM1* oncogenic interaction and limiting drug efficacy, received much criticism. Since it has been reported that prolonged (in contrast to intermittent) XPO1 inhibition down-regulates the HOX/MEIS axis, inducing the differentiation of the *NPM1*-mutated AML cells, the more frequent administration of the second-generation XPO1 inhibitor eltanexor (KPT-8602) has been proposed. Eltanexor prolonged the survival of leukemic mice, caused HOX down-regulation and induced AML differentiation in [103]. Moreover, eltanexor has the advantage of lower central nervous system (CNS) penetration with less anorexia, permitting frequent dosing and higher drug concentration, resulting in the stable inhibition of the *NPM1*–XPO1 interaction and promising activity preclinically [109].

Interestingly, the combination of the known B-cell lymphoma 2 (BCL2) inhibitor VEN with SINEs has shown important efficacy in preclinical models [110] and is being evaluated currently in clinical trials (NCT03955783). Even though the full mechanism of MCL1-mediated synergy between VEN and selinexor or eltanexor remains unknown, it is more likely that VEN enhances the selinexor- and eltanexor-induced DNA damage of leukemic cells, mainly through inhibiting DNA damage repair [111]. Furthermore, VEN exerts its anti-leukemic action against AML cells in synergy with selinexor in vitro by inhibiting the glycolytic leukemic cell function, along with down-regulating the DNA replication-related genes [112]. The relevant synergistic clinical trials are eagerly anticipated.

### 4.2. NPM1-Mutated AML and Menin-KMT2A Inhibitors

*NPM1*-mutated AMLs overexpress the HOX gene (HOXA9) and activate the oncogenic axis HOXA9/MEIS/CCAAT enhancer-binding protein alpha (CEBPA)/lysine methyl transferase 2 (KMT2A) [104,106,107]. KMT2A is a transcriptional regulator, binding to menin and forming the menin–KMT2A oncogenic complex, which alters HOXA9 expression [104,113,114]. As *NPM1*-mutated AMLs overexpress the menin-KMT2A-HOX axis, the inhibition of menin-KMT2A could potentially serve as a therapeutic target for this leukemic subset. The latter inhibition has shown efficacy in animal models [115]. In conclusion, cytoplasmic-mutated *NPM1* is highly dependent on the menin–MLL interaction. Pre-leukemic and leukemic *NPM1*-mutated cells can also be eradicated in vivo by menin inhibitors. The links between cytoplasmic mutated *NPM1* and chromatin and between cytoplasmic mutated *NPM1* and MLL1 remain undetermined.

Revumenib (SNDX-5613), a menin inhibitor, has shown a 60% overall response rate (ORR) in *KMT2A*- or *NPM1*-mutated AML. The early phase I/II study (AUGMENT-101, NCT04065399) involved heavily pretreated AML patients [relapsed/refractory (R/R) AML] [116]. The *NPM1*-mutated AMLs were 21% of the whole study cohort, whereas 68% of the patients were diagnosed with a KMT2A-r AML. SNDX-5613 was administered orally, Q12h, in 28-day cycles. Grade 2 acute differentiation syndrome was observed in 16% of the patients and resolved with steroids and/or hydroxyurea. A grade 3 QTc prolongation was also evident in a subset of the patients [116]. These promising results were also confirmed by a longer follow-up in the AUGMENT-101 phase 2 study of revumenib in patients with R/R KMT2Ar acute leukemias [117].

Based on the results of the AUGMENT-101 clinical trial, the Food and Drug Administration (FDA) approved revumenib on 15 November 2024 in the United States for treating relapsed/refractory acute leukemia with a *KMT2A* translocation in adult and pediatric patients aged 1 year old and older [118]. This oral drug has not yet been approved in Europe. *KMT2A* abnormalities are observed in 5–15% of the cases of acute lymphoblastic leukemia (ALL) and in 3% of adult patients with AML [119]. Revumenib addresses a challenging subset of leukemias, often resistant to standard treatment. The drug achieves deep responses, maintains remission after HSCT and has a manageable safety profile (Table 2) [118].

The safety and activity of revumenib in combination with fludarabine/cytarabine (FLA) in patients with relapsed/refractory acute leukemias has been evaluated recently with encouraging results (NCT05326516) (Table 2) [120]. Furthermore, the phase I/II study of the oral combination of revumenib with Decitabine/Cedazuridine (ASTX727) and VEN (SAVE) in R/R AML is underway (NCT05360160) (Table 2) [127]. Finally, the following interesting, randomized phase III clinical trial will compare revumenib plus VEN-AZA with placebo plus VEN-AZA in *NPM1*-mutated or *KMT2A*-rearranged AML patients (NCT06652438).

Ziftomenib (KO-539) is another menin inhibitor under evaluation in the phase I/II clinical trial KOMET-001 (NCT04067336) for treating R/R AML patients [130]. Ziftomenib was dosed orally, once daily, in 28-day cycles, and the optimal dose was found to be 600 mg. A CR of 30% and an ORR of 40% were demonstrated in 20 patients with R/R *NPM1*-mutated AML [130]. No QTc prolongation related to the drug was observed. Acute differentiation syndrome and lung inflammation were manageable adverse events and well tolerated. Ziftomenib seems to be a promising agent for *KMT2A*- or *NPM1*-mutated AML, and ongoing evaluation is necessary in future clinical trials [130]. The interim phase 1a results from the KOMET-007 study regarding the application of Ziftomenib combined with VEN/AZA in R/R *KMT2A* or *NPM1*-mutated AML (NCT05735184) are shown in Table 2 [121]. Ziftomenib can also be combined with intensive induction (7 + 3) in ND *KMT2A*- or *NPM1*-mutated AML (NCT05735184) [131].

Other menin inhibitors such as enzomenib (DSP-5336) or bleximenib are currently being tested in *KMT2A* or *NPM1*-mutated AML. The phase 1 results of the use of enzomenib in patients with R/R acute leukemia are presented in Table 2 (NCT04988555) [125]. The specific chemical properties of enzomenib compared to other menin inhibitors minimize off-target toxicity. Bleximenib combined with intensive chemotherapy in ND AML with *KMT2Ar* or *NPM1* alterations is evaluated in a phase 1b study (NCT05453903) (Table 2) [126]. A bleximenib RP2D dose of 100 mg twice daily with optimal safety has been recently demonstrated [132]. A phase II study of bleximenib monotherapy (NCT04811560, namely a cAMeLot-1 study) in R/R AML patients with *KMT2Ar* or *NPM1* alterations is ongoing [132]. Moreover, a phase III, randomized, double blind, placebo-controlled clinical trial, cAMeLot-2, will evaluate the combination of bleximenib, VEN and AZA in AML patients (NCT06852222, Table 3). In general, the toxicity of menin inhibitors involves differentiation syndrome and QTc prolongation in all clinical trials and all tested lines of therapy, and they are well-known complications treated by physicians [118,125,126,130].

Finally, in an important work, Uckelmann HJ et al. described the crucial functional role of mutant *NPM1* as a direct driver of oncogenic gene expression in AML [133]. They highlighted that cytoplasmic *NPM1* binds to chromatin and co-operates with the MLL complex. This provides functional insight into the mechanism of menin–MLL inhibition, which disrupts the binding between *NPM1* and chromatin, in *NPM-1*-mutated AMLs [37,38,133].

### 4.3. NPM1-Mutated AML, Knockout and Knock-In Phenotype

The complete pathophysiological linkage between menin or exportin-1 inhibitors and *NPM1* mutants remains largely unknown. The knockout (KO) of mutant *NPM1* from AML cells, using CRISPR-Cas9 gene editing, significantly eliminates the sensitivity of the leukemic cells to menin, exportin-1 inhibitors and cytarabine [113], indicating the presence of a therapeutic window for these substances. On the other hand, the knock-in of a copy of mutant *NPM1* sensitizes AML cells to treatment with cytarabine or menin inhibitors (revumenib, ziftomenib). Furthermore, treatment with pan-HDAC inhibitors (panobinostat) or WEE1 tyrosine kinase inhibitors (adavosertib) exhibits synergistic in vitro action with menin inhibitors in *NPM1*-mutated AMLs. The latter combination also shows important efficacy in AML xenograft models [113].

### 4.4. NPM1-Mutated AML, All-Trans Retinoic Acid (ATRA) and Arsenic Trioxide (ATO)

The anti-leukemic activity of all-trans retinoic acid (ATRA) in combination with chemotherapy in non-acute promyelocytic leukemia (APL) has been initially demonstrated in vitro. ATRA enhances the cytotoxic effect of cytarabine or idarubicin when administered sequentially, after the cytotoxic drug [134,135]. This sequence-dependent synergy may partly be attributed to the reduction in BCL2’s half-life, a mechanism also implicated in drug resistance in AML [134,135]. Notably, in *NPM1*-mutated AML, ATRA decreases *NPM1* protein levels by selectively inducing apoptosis and sensitizing leukemic cells to cytarabine [136]. Furthermore, the combination of ATRA and arsenic trioxide (ATO) synergistically promotes the proteasomal degradation of the mutant *NPM1* protein with an unclear mechanism, resulting in leukemic cell differentiation, growth inhibition and apoptosis in *NPM1*-mutated AML treated with daunorubicin [137,138,139].

It has been proposed that the synergy of ATRA and ATO reverses the characteristic disorganization of promyelocytic leukemia (PML) bodies, as also observed in all types of *NPM1*-mutated AML, apart from APL. ATRA and ATO induce oxidative stress in the leukemic cells, in parallel with the non-specific disruption of the stress-related pathways required for oncoprotein maintenance in *NPM1*-mutated AML [139].

Nevertheless, data from large, randomized trials exploring the administration of ATRA combined with intensive or non-intensive chemotherapy in *NPM1*-mutated AML yielded conflicting results. Some studies demonstrated clinical benefit, whereas others reported no significant impact of the drug. In the UK MRC AML12 trial, adding ATRA to induction chemotherapy in young patients (15–60 years) with *NPM1*-mutated AML showed no significant survival benefit [140]. Moreover, the results of the randomized AMLSG 15–10 trial exhibited an inferior outcome and decreased OS for the ATRA arm in combination with low-dose cytarabine plus etoposide in older patients (>60 years) with ND *NPM1*-mutated AML, making it unfit for intensive chemotherapy [123]. Contrarily, in the AMLSG 07-04 trial, adding ATRA to standard induction therapy in younger adults (18–60 years) with *NPM1*-mutated/FLT3-ITD-negative AML led to improved EFS and OS [141]. Similarly, adding ATRA to induction and consolidation therapy in elderly AML patients (>60 years old) improved CR rates, EFS and OS [142]. Despite the heterogeneous results, these studies highlight the biological and therapeutic rationale of ATRA in *NPM1*-mutated AML, supporting the continued investigation of ATRA/ATO-based strategies, possibly in combination with other targeted therapies, in this AML molecular subtype.

### 4.5. NPM1-Mutated AML and Vitamin C and D Supplements

The role of vitamin C and D supplementation in AML has recently been investigated. Patients benefiting the most from vitamin C and D supplementation were those diagnosed with *NPM1*-mutated AML in a recent study [143]. These vitamins are usually administered as supportive care from day 10 of AML induction chemotherapy until hematologic recovery from induction and consolidation treatments. Interestingly, during AML induction, a lower incidence of complications including macrophage activation syndrome or hemorrhage and a significantly lower rate of bacterial and fungal infections were observed in the vitamin C and D arms for all AML patients.

Moreover, while the aforementioned study showed no difference in OS between those who received supplementation and those who did not receive it, a subgroup analysis yielded that the risk of death was, surprisingly, nearly 50% lower among those who were positive for the *NPM1* mutation and receiving vitamin C and D supplements during treatment [143]. Based on the above finding, vitamin C and D supplementation is recommended in AML patients harboring *NPM1* mutations for improving their OS and in all AML patients for lowering the rates of grade 3 and 4 adverse events [143]. The possible anti-leukemic mechanisms of vitamin C and D still need to be fully elucidated.

## 5. Optimal Treatment Approach for *NPM1*-Mutated AML in 2025

### 5.1. General Treatment Decisions and Standard Chemotherapy Regimens

Patients with an *NPM1* mutation in the absence of *FLT3-ITD* are classified in the favorable prognostic risk category. Those who are fit for intensive chemotherapy will receive induction treatment intravenously, usually a combination of cytarabine in a continuous 7-day infusion (100–200 mg/m^2^/day) with an anthracycline (either daunorubicin 60 mg/m^2^ or idarubicin 12 mg/m^2^) for 3 days (7 + 3 regimen), depending on the protocol applied at each center. In this subset of patients, HSCT in CR is not recommended due to the favorable prognosis [81]. After CR, these patients receive consolidation therapy. Maintenance therapy with oral AZA (CC-486) can be administered as monotherapy after induction treatment (with or without consolidation) for AML patients who will not be transplanted [144]. The unfit patients of the above prognostic category or those >75 years old will receive a combination of VEN plus HMA [85,86].

On the contrary, patients with an *NPM1* mutation in the presence of *FLT3-ITD* are classified in the intermediate prognostic risk category [31], and eligible patients will undergo allogeneic HSCT after induction and/or consolidation treatment. Interestingly, AML patients with an *NPM1* and a *WT1* mutation belong to the adverse prognostic group [88]. Thus, a patient with an *NPM1* mutation can even belong to the adverse group. This is also the case when the *NPM1* mutation and adverse-risk cytogenetic abnormalities co-exist. *NPM1*-mutated AMLs with an *FLT3-ITD* mutation should also receive midostaurin (50 mg of q12h per OS) during days 8–21 of induction and in all cycles of consolidation [87]. Unfit patients at intermediate or adverse risk with *NPM1*-mutated AML or those >75 years old should also receive VEN plus HMA [85,86]. VEN-based combination therapies are a promising targeted approach for *NPM1*-mutated AML because they have been linked with durable molecular remission and increased OS [91,145].

MRD assessment should be performed after two cycles of chemotherapy and at the end of treatment [31]. There is a correlation between MRD positivity and the risk of relapse. AML patients who are MRD-positive at the end of consolidation benefit from allogeneic HSCT regarding OS. Without HSCT, they will relapse. Before transplantation, MRD evaluation should also be conducted, because positivity has been associated with poor outcomes even after HSCT [146].

Because increased CD33 expression is observed in blastic cells of *NPM1*-mutated AML, the anti-CD33 monoclonal antibody GO (3 mg/m^2^) is used in younger fit adults with this AML subtype [82,147]. This antibody is chemically linked to a calicheamicin-based cytotoxic warhead. A meta-analysis showed a clinical benefit for GO in frontline AML therapy, especially in patients with core binding factor (CBF) AML [84]. Furthermore, a reduction in the probability of relapse and greater molecular clearance of the *NPM1* mutants, albeit with EFS difference, has been observed in *NPM1*-mutated AML [83,148]. According to these findings, based on the National Comprehensive Cancer Network (NCCN) guidelines, GO is a category 2B recommendation option for favorable-risk AMLs, such as *NPM1*-mutated AML. The use of GO is, therefore, recommended on days 1, 4 and 7 of induction and on day 1 of consolidation, but a single dose on day 1 of induction may also be effective [84,149,150].

The optimal number of consolidation cycles is unknown. There is a lack of prospective data supporting the four-cycle standard of care. The current opinion is that the number of cycles is linked to the dose of cytarabine. The European Leukemia Net (ELN) 2022 guidelines recommend administering three to four cycles of intermediate-dose cytarabine (IDAC, 1–1.5 g/m^2^ every 12 h on days 1, 2 and 3), which can be reduced to 0.5–1 g/m^2^ in AML patients older than 60 years old [31]. The number of cycles required for applying IDAC (1–1.5 g/m^2^) is a matter of debate, as insufficient evidence-based data exist. A decrease to two IDAC cycles is recommended for elderly patients. Therefore, either a dose reduction (0.5–1 g/m^2^, 3–4 cycles) or a cycle reduction (1–1.5 g/m^2^, 2 cycles) is usually applied for older AML patients [151].

The Hellenic AML National protocol (AML-HSH-2019) for AML patients aged 15–65 years old, eligible for intensive chemotherapy, recommends three consolidation cycles of IDAC (1–1.5 g/m^2^) in most patients after two cycles of induction treatment. An exception is the application of HiDAC (3 g/m^2^) in all patients < 45 years old with core binding factor (CBF) leukemias [152]. Additional dose modifications for cytarabine for the second and third consolidation cycles are applied based on the age of the patient for CBF (3 g/m^2^ < 45 years old, 1.5 g/m^2^ 45–60 years old, 1 g/m^2^ > 60 years old) and *NPM1*-mutant (1.5 g/m^2^ < 60 years old, 1 g/m^2^ > 60 years old) AMLs. Moreover, a cycle of GO (3 g/m^2^), along with cytarabine (IDAC, 1–1.5 g/m^2^) for only the second induction and the first consolidation cycle, is recommended for patients with CBF or *NPM1*-mutant AML (the protocol is a close variation of the ELN recommendations of that time [153]).

The optimal treatment for an *NPM1*-mutated AML patient according to the mutational pattern is shown in Table 1.

### 5.2. Emerging Clinical Trials for NPM1-Mutated AML

A list of 35 clinical trials (8 randomized) is shown in Table 3 (updated 1 June 2025). Interestingly, two thirds of these clinical trials (23/35) involve menin inhibitors, either alone or in combination with other drugs, which is a rapidly evolving field in *NPM1*-mutated AML. The clinical trials with the most promising clinical responses in patients with *NPM1*-mutated AML are shown in Table 2.

The phase III, randomized, two-arm, open-label study of chemotherapy in combination with ATRA with or without GO in patients with *NPM1*-mutated AML (NCT00893399) yielded a statistically significant relapse reduction with GO (Table 2) [122]. Another phase III, randomized, two-arm study investigated the use of low-dose cytarabine and etoposide with or without ATRA in older patients not eligible for intensive chemotherapy with AML and *NPM1* mutation (NCT01237808, Table 2) [123]. The retrospective observational clinical study conducted by Orvain C et al. evaluated the outcomes of patients with CBF leukemias and/or *NPM1*-mutated AML after first-line intensive chemotherapy in their first molecular relapses (NCT04931992, Table 2) [124]. Promising results that render the combination of VEN-AZA as a bridge to transplant therapy have been documented in the single-arm, phase II, non-randomized study of VEN and AZA for managing molecular relapse/progression in adult *NPM1*-mutated AML (NCT04867928, Table 2) [129,154]. Finally, the phase II results of the randomized study comparing VEN-AZA and intensive chemotherapy in fit patients with ND *NPM1*-mutated AML (VINCENT study, NCT05904106, Table 2) are eagerly anticipated [128].

## 6. Immunotherapies—Immunomodulatory Drugs

### 6.1. Checkpoint Inhibitors

Immune checkpoint molecules, such as PD-1 and its ligand PD-L1, play a pivotal role in leukemogenesis by fostering an immunosuppressive tumor microenvironment. The signal transducer and activator of transcription 5 (STAT5) is overexpressed in AML and activates the promoter of glycolytic genes to enhance glycolysis in leukemic cells. Thus, increased lactate accumulation promotes histone lactylation, thereby inducing PD-L1 transcription, another leukemogenic mechanism [155]. The presence of *NPM1* mutations in AML appears to enhance responsiveness to PD-1-based immunotherapies, suggesting that the underlying genetic alterations of the disease might critically influence the effectiveness of checkpoint blockade [156,157].

Nivolumab, a monoclonal antibody targeting PD-1, has shown promising results in preclinical and early-phase clinical trials by boosting the cytotoxic activity of T cells against leukemic cells harboring *NPM1* mutations. These findings suggest that the inhibition of the PD-1 signaling pathway can restore the function of exhausted T cells, thereby enhancing the immune system’s ability to eliminate leukemic progenitor cells [156,158].

Although PD-1 inhibition alone has shown limited clinical efficacy in AML, combining PD-1 blockade with other agents may enhance both anti-leukemic and immunostimulatory responses. Notably, AZA induces the up-regulation of PD-1 and PD-L1 expression in AML cells, a phenomenon linked to the development of resistance to AZA treatment. This mechanism of immune escape might be overcome by targeting PD-1 with checkpoint inhibitors, such as nivolumab and pembrolizumab [157,159].

Pembrolizumab has been evaluated mainly in combination with HMAs, such as AZA. This combination can induce molecular remission in a subset of patients with relapsed *NPM1*-mutated AML (NCT02845297). Furthermore, the combination of nivolumab and AZA with or without the monoclonal antibody Ipilimumab has been tested in patients with R/R or ND AML, including patients harboring *NPM1* mutations, with variable responses and outcomes, along with significant toxicity (NCT02397720).

The first randomized study of an anti-PD1 antibody (pembrolizumab) added to AZA+VEN in ND AML patients ineligible for induction therapy highlighted significant toxicity. The addition of pembrolizumab to AZA plus VEN did not improve MRD-negative CR/CRi and was associated with a trend for worse outcomes (NCT04284787) [160].

In conclusion, checkpoint blockade offers a novel therapeutic avenue in *NPM1*-mutated AML, but further clinical trials are essential for optimizing treatment protocols, identifying predictive biomarkers and establishing long-term benefits for patients.

### 6.2. Lenalidomide

Lenalidomide exerts its anti-leukemic actions by activating T and natural killer (NK) cells and through inhibiting proinflammatory cytokines, such as interleukin-6 (IL-6) and tumor necrosis factor-alpha (TNF-α). In general, the drug has anti-proliferative, anti-angiogenetic, anti-inflammatory and pro-apoptotic capacities. It also enhances anti-tumor immunity [161]. A 30% response rate has been reported for lenalidomide as a first-line treatment in older ND AML patients [162]. However, the HOVON-SAKK-132 clinical trial found limited therapeutic benefit, especially in younger and middle-aged adults [163].

Lenalidomide could be used as a potential treatment for *NPM1*-mutated AML, especially when combined with checkpoint inhibitors to improve leukemia-associated antigen-specific response [164]. When both these drugs are used alone as standard induction therapy, they do not have a significant effect, unless other requirements are met; the *SRSF2* genotype is used for lenalidomide- [163] or biomarker-driven approaches for checkpoint inhibitors [164]. Despite the promising effect of this treatment combination, limitations and questions still arise, requiring further preclinical and clinical evaluation, along with new promising treatment combination testing. Interestingly, the immunomodulatory agent mezigdomide has shown efficacy alone and in combination with menin inhibition in preclinical models of *KMT2Ar*- and *NPM1*-mutated AML [165].

### 6.3. Imiquimod Analogs

Imiquimod analogs, particularly imiqualine, have emerged as promising agents in treating *NPM1-mutated AML*. The imiqualine EAPB0503 has shown selective potency, inducing apoptosis. The molecule promotes the proteasomal-mediated degradation of mutant *NPM1*, restoring the nuclear localization of normal *NPM1* and suppressing leukemic cell proliferation [166].

Recently, a second-generation imiqualine, EAPB02303, has shown 200-fold greater potency and broader activity across AML subtypes, exhibiting selective efficacy against *NPM1*-mutated AML, making it a promising candidate for further clinical evaluation [167].

## 7. Unmet Needs—CAR-T Cells: Proposed Future Directions

There is an unmet need for novel investigational agents or combinations of novel agents with already well-established drugs in *NPM1*-mutated AMLs. The detailed description of chimeric antigen receptor (CAR)-T and T-cell receptor (TCR) therapies are investigational approaches that are beyond the scope of this review.

Due to the lack of selective target antigen overexpression on AML blasts, along with the required absence of the same antigen on normal tissues, there are hurdles limiting the clinical application of CAR-T cell therapies in AML patients, as non-specific targeting causes severe toxicities [168,169]. The cytoplasmic *NPM1*–HLA complex can be targeted by anti-NPM1c/HLA-A2 CAR-T or memory NK cells [170,171,172]. An important limitation of this strategy is the difficulties in triggering full T-cell stimulation. Constructing dual CAR-T cells that co-express an anti-NPM1c/HLA-A2 CAR and an anti-CD123 co-stimulatory receptor (CCR) without activating the signaling domain might offer a possible solution to the problem. CD123 is highly expressed in *NPM1*-mutated AML [173,174]. Moreover, Van der Lee et al. successfully transduced CD8+ and CD4+ T cells with specificity for *NPM1*-mutated peptides, targeting HLA A2-restricted primary leukemic blasts [172,175].

While CAR-T cell therapy is still in its early stages in AML, cytotoxic T cells may help in maintaining remission and achieving prolonged survival. They could also play a role in eradicating residual disease, especially for patients ineligible for allogeneic HSCT. In conclusion, ongoing research is essential for deciphering the complex pathophysiology of the *NPM1* mutation in AML, which will lead to novel targeted therapies.

## Figures and Tables

**Table 1:** Optimal treatment for an NPM1-mutated AML in 2025 based on the mutational patient profile. The reader should see the detailed text for dosing regimens and number of cycles for induction, consolidation and the use of gemtuzumab ozogamicin (GO). The question mark (?) refers to combinations of mutations where there is no consensus regarding the prognostic classification.

Mutations/Prognostic Risk Group	Treatment
**Fit:** *NPM1^mut^/FLT-ITD^wt^/DNMT3A^wt^/WT1^wt^* *NPM1^mut^/IDH1^mut^ ?* *NPM1^mut^/DNMT3A^mut^/NRAS^G12/13-mut^* *NPM1^mut^*/*IDH2^mut^* *NPM1^mut^/FLT-ITD^mut^/cohesin^mut^* *NPM1^mut^/NRAS^mut^/cohesin^mut^* *Döhner H et al., Blood 2022 (ELN)* [31] ***Favorable*** **Unfit:** *NPM1^mut^/FLT-ITD^wt^/NRAS^wt^/KRAS^wt^/TP53^wt^* *Döhner H et al., Blood 2024 (ELN)* [78]	**Fit:** Intensive chemotherapy Induction: 7 + 3 regimen Consolidation treatment No HSCT Gemtuzumab Ozogamicin—GO (either in induction and/or consolidation depending on the applied protocol) *Pratcorona M et al., Blood 2013* [81] *Castaigne S et al., Lancet 2012* [82] *Schlenk RF et al., J Clin Oncol 2020* [83] *Hills RK et al., Lancet Oncol 2014* [84] *Döhner H et al., Blood 2022 (ELN)* [31] **Unfit or >75 yrs:** Venetoclax + HMA *DiNardo CD et al., N Engl J Med 2020* [85] *Pratz KW et al., Blood 2022* [86]
**Fit:** *NPM1^mut^/FLT-ITD^mut^* *NPM1^mut^/FLT-ITD^mut^/DNMT3A^mut^ ?* *NPM1^mut^/IDH1^mut^ ?* *Döhner H et al., Blood 2022 (ELN)* [31] ***Intermediate*** **Unfit:** *NPM1^mut^/FLT-ITD^mut^* *NPM1^mut^/KRAS^mut^* *NPM1^mut^/NRAS^mut^* *Döhner H et al., Blood 2024 (ELN)* [78]	**Fit:** Intensive chemotherapy Induction: 7 + 3 regimen plus Midostaurin (d. 8–21 of induction) Consolidation treatment plus Midostaurin (all cycles of consolidation) HSCT *Stone RM et al., N Engl J Med 2017* [87] *Döhner H et al., Blood 2022 (ELN)* [31] **Unfit or >75 yrs:** Venetoclax + HMA *DiNardo CD et al., N Engl J Med 2020* [85] *Pratz KW et al., Blood 2022* [86]
**Fit:** *NPM1^mut^/WT1^mut^* *NPM1^mut^/adverse risk cytogenetics* *NPM1^mut^/FLT-ITD^mut^/DNMT3A^mut^ ?* *Döhner H et al., Blood 2022 (ELN)* [31] ***Adverse*** **Unfit:** *TP53^mut^* *Döhner H et al., Blood 2024 (ELN)* [78]	**Fit:** Intensive chemotherapy Induction: 7 + 3 regimen plus Midostaurin (d. 8–21 of induction) Consolidation treatment plus Midostaurin (all cycles of consolidation) HSCT *Stone RM et al., N Engl J Med 2017* [87] *Döhner H et al., Blood 2022 (ELN)* [31] **Unfit or >75 yrs:** Venetoclax + HMA *DiNardo CD et al., N Engl J Med 2020* [85] *Pratz KW et al., Blood 2022* [86]

**Table 2:** Clinical trials involving AML patients with NPM1-mutated AML with promising clinical responses. Regarding toxicity, grade ≥ 3 is provided, unless otherwise stated for any-grade side effects. Abbreviations: AE: adverse events; ALL: acute lymphoblastic leukemia; allo-HCT/alloSCT: allogeneic hematopoietic cell/stem cell transplantation; AML: acute myeloid leukemia; ATRA: all-trans retinoic acid; AZA: azacitidine; BID: bis in die (twice daily); CI: confidence interval; CR: complete remission; CRc: composite complete remission; CRh: complete remission with partial hematologic recovery; CRi: complete remission with incomplete hematologic recovery; CTCAE: Common Terminology Criteria for Adverse Events; DA: Daunorubicin + Cytarabine; DL: dose level; DS: differentiation syndrome; ETO: Etoposide; FLA: Fludarabine + Cytarabine; FLT3-ITD: FMS-related tyrosine kinase 3—internal tandem duplication; GO: Gemtuzumab Ozogamicin; ITT: intention to treat; IV: intravenously; MRD: measurable residual disease; ND: newly diagnosed; NR: not reached; ORR: overall response rate; OS: overall survival; PO: per os; PS: performance status; pts: patients; q12h: every 12 h (twice daily); QD: quaque die (once daily); RP2D: recommended phase 2 dose; RR: response rate; R/R: relapsed/refractory; SC: subcutaneously; SOC: standard of care; VEN: Venetoclax.

Reference	Trial Phase	Title of the Trial	Targeted Drugs—Dose	Study Population	Response Rates	Grade 3–4 Toxicity	Study Status	Clinical Trial Identifier
Shukla N et al., HemaSphere 2024 [120]	Phase I, non- randomized, open-label, interventional, dose- escalation, parallel assignment	A study of Revumenib in combination with chemotherapy in participants with relapsed/refractory acute leukemia (AUGMENT-102)	Revumenib: 113–163 mg/dose, per os, q12h × 28d FLA chemotherapy: – Fludarabine: 30 mg/m^2^ IV, days 1–5 – Cytarabine: 2000 mg/m^2^ IV, days 1–5	ITT: 27 pts DL1: 9 pts DL2: 18 pts Median age: 6 y (range: 0.8–78) <18 y: 20 pts (74%) <2 y: 5 pts (19%) KMT2Ar: 24 pts (89%) NPM1m: 2 pts (7%) NUP98r: 1 pt (4%) ≥3 prior therapies: 19 pts (70%) Prior HSCT: 11 pts (41%)	DL1 (n = 9): CRc: 4 pts (44%) Stable disease: 1 pt (11%) Progressive disease: 2 pts (22%) Death before assessment: 2 pts (22%) DL2 (n = 18): CRc: 9 pts (50%) Stable disease: 6 pts (33%) Progressive disease: 3 pts (17%) MRD negativity in CRc pts: 12/13 (92%) Median time to response: 1.0 mo (DL2) Median duration of response: Not reached (95% CI: 9.2 mo – NR)	Grade 3 ALT ↑ (DL1) Grade 4 neutropenia (DL2, post-HSCT pt) QTc prolongation (≥grade 2): 4 pts (15%) No grade 3 QTc prolongation No differentiation syndrome (DS) 1 death due to sepsis (DL1) Most common AEs (>50%): anemia, thrombocytopenia	Completed	NCT05326516
Fathi AT et al., Blood 2024 [121]	Phase I, non- randomized, interventional, open- label, parallel assignment	A study to investigate the safety and tolerability of Ziftomenib in combination with venetoclax/azacitidine, venetoclax, or 7 + 3 in patients with AML: Ziftomenib/VEN/AZA (R/R) Ziftomenib/7 + 3 (first line) Ziftomenib/VEN/AZA (first line) Ziftomenib/VEN (R/R) (KOMET-007)	Ziftomenib: 200/400/600 mg, per os, once daily, from Day 8 onwards VEN: per label; days 1–28, per os AZA: 75 mg/m^2^, SC or IV; days 1–7/28-day cycle	ITT: 54 pts NPM1-m cohort: 26 pts KMT2A-r cohort: 28 pts Median age: 53 years (range: 22–86) Female: 56% Prior venetoclax: 69% Prior HSCT: 30% Menin inhibitor-experienced: 20% Median prior therapies: 2 (range: 1–8)	NPM1-m (n = 22): ORR: 68% CRc: 50% Median duration of CRc: 23.4 weeks 6-mo OS rate: 77% KMT2A-r (n = 27): ORR: 33% CRc: 15% Median duration of CRc: not reached 6-mo OS rate: 43%	Any Grade ≥ 3 AEs (all patients): – Platelet count ↓: 31% – Anemia: 26% – Febrile neutropenia: 26%	Recruiting	NCT05735184
Döhner H et al., Lancet Haematol 2023 [122]	Phase III, randomized, two-arm, open-label, interventional	Study of chemotherapy in combination with all-trans retinoic acid (ATRA) with or without gemtuzumab ozogamicin in patients with acute myeloid leukemia (AML) and mutant nucleophosmin-1 (NPM1) gene mutation: chemotherapy with ATRA and GO vs. chemotherapy with ATRA without GO	GO: 3 mg/m^2^, IV; Day 1 of induction cycles 1 and 2 + consolidation cycle 1 IDA: 12 mg/m^2^, IV; Days 1, 3, 5 (Cycle 1), Days 1, 3 (Cycle 2) Ara-C: 100 mg/m^2^, IV, Days 1–7 ETO: 100 mg/m^2^, IV; Days 1–3 (reduced to 2 days in Cycle 2) ATRA: 45 mg/m^2^ PO; Days 6–8 → 15 mg/m^2^ PO; Days 9–21 HDAC (consolidation): 3000 mg/m^2^ q12h; Days 1–3 (1000 mg/m^2^ if >60 yrs)	ITT: 588 pts – GO group: 292 pts – Control group: 296 pts Median age: 58.7 yrs Female: 54% FLT3-ITD+: 17%, DNMT3A+: 51% De novo AML: 93% Favorable ELN risk: 90%	CR/CRi: 90% (control) vs. 86% (GO) CR: 58% (control) vs. 47% (GO) CR/CRh: 72% (control) vs. 67% (GO) 2-year OS: 69% (control) vs. 73% (GO) 2-year CIR: 37% (control) vs. 25% (GO) Statistically significant relapse reduction with GO (*p* = 0.0028)	Most common Grade 3–4 AEs (GO group): Febrile neutropenia: 44% Thrombocytopenia: 90% Pneumonia: 25% Sepsis: 29% Grade 5 AEs (GO group): – Deaths from sepsis/infection: 6%	Completed	NCT00893399
Schlenk R et al., Scientific Reports 2023 [123]	Phase III, randomized, two-arm, open-label, interventional, multicenter	Study of low-dose cytarabine and etoposide with or without all-trans retinoic acid in older patients not eligible for intensive chemotherapy with acute myeloid leukemia and NPM1 mutation: low-dose cytarabine- etoposide without ATRA vs. low-dose cytarabine-etoposide with ATRA	ATRA: 45 mg/m^2^ PO; days 8–28 → reduced to 45 mg/m^2^ days 8–10, then 15 mg/m^2^ days 11–28 Cytarabine: 20 mg/day SC, BID; days 1–7 (per cycle) Etoposide: 50 mg/m^2^/day IV, days 1–3 (Cycle 1); → 100 mg/day PO/IV; days 1–3 (Cycles 2–6)	ITT: 144 pts – ATRA arm: 72 pts – Control arm: 72 pts Median age: 76.8 yrs (range: 63.8–91.8) Male: 51.4% FLT3-ITD+: 27.1% De novo AML: 87.5%	CR/CRi: 33.3% (ATRA) vs. 36.1% (CONTROL) Median OS: 5.0 mo (ATRA) vs. 9.2 mo (CONTROL); *p* = 0.023 2-yr OS rate: 7% (ATRA) vs. 10% (CONTROL)	Infections (Cycle ≥ 2): 59% (ATRA) vs. 32% (CONTROL); *p* = 0.01 Early death (first 2 cycles): 18.1% (ATRA) vs. 8.5% (CONTROL); *p* = 0.09 Gastrointestinal AEs ≥ grade 3: 6 pts (ATRA) vs. 4 pts (CONTROL) Pulmonary AEs ≥ grade 3: 5 pts (ATRA) vs. 0 pts (CONTROL) No significant differences in CTCAE grade 3–4 toxicities during Cycle 1	Completed	NCT01237808
Orvain C et al., Leukemia 2024 [124]	Retrospective, observational	Outcome of patients with CBF and/or NPM1- mutated AML in first molecular relapse	Induction therapy: – Daunorubicin: 60–90 mg/m^2^, IV, days 1–3 – Idarubicin: 8–9 mg/m^2^, IV, days 1–5 – Cytarabine: 200 mg/m^2^, IV, days 1–7 Consolidation: – Intermediate/high-dose cytarabine: 1.5–3 g/m^2^/12 h × 3 days Salvage therapy after relapse – Upfront allo-HCT: 15% – Intensive chemotherapy: 63% – non-intensive (e.g., AZA, VEN, FLT3/IDH inhibitors): 20%	ITT: 303 pts – CBF AML: 47% – NPM1-mutated AML: 53% – Age: 18–60 yrs (median ~47 yrs) – No allo-HCT in 1st CR – Monitored for MRD after intensive therapy Relapse categories: – No relapse: 153 pts (51%) – Molecular relapse: 95 pts (31%) – Upfront morphologic relapse: 55 pts (18%)	3-year OS (based on relapse type): – Preemptive therapy (molecular relapse): 78% –Molecular→Morphologic relapse: 52% – Upfront morphologic relapse: 51% *p* = 0.02 Preemptive therapy subgroups (3-year OS): – Upfront allo-HCT: 92% – Intensive chemotherapy: 79% – Non-intensive therapy: 58% *p* = 0.09	Specific grade 3–4 AE rates not numerically detailed	Unknown	NCT04931992
Zeidner JF et al., Blood 2024 [125]	Phase I, II, open-label, interventional, dose- escalation, dose- expansion, non- randomized	A study of Enzomenib (DSP-5336) in relapsed/refractory AML/ALL with or without MLL rearrangement or NPM1 mutation	Enzomenib (DSP-5336): – Oral administration, BID (twice daily) – Dose range: 40 mg → 300 mg BID – Active dose: ≥140 mg BID	ITT: 81 patients – Arm A: 31 pts – Arm B: 50 pts	Patients treated with active doses (≥140 mg BID) and no prior menin inhibitor: KMT2Ar (n = 22): – ORR (CR + CRi + MLFS): 59.1% – CR + CRh: 22.7% NPM1m (n = 13): – ORR: 53.8% – CR + CRh: 23.1% Other mutations: – 1 AML pt achieved CR Median time to response: – ORR and CR + CRh: 1.0 month	Treatment-related AEs ≥10%: – Vomiting: 14.8% – Nausea: 13.6% – Only 1 pt had grade 3 vomiting/nausea Any-grade AEs ≥ 20%: – Nausea: 39.5% – Vomiting: 29.6% – Febrile neutropenia, diarrhea, hypokalemia: 22.2% – Appetite loss, headache: 21.0%	Recruiting	NCT04988555
Recher C et al., Blood 2024 [126]	Phase Ib, interventional, non-randomized, 3 arms	A study of bleximenib (JNJ-75276617) in combination with acute myeloid leukemia (AML) directed therapies: Arm A (R/R): JNJ-75276617, VEN, AZA Arm B (ND, chemo-ineligible): JNJ-75276617, VEN, AZA Arm C (ND, chemo-eligible): JNJ-75276617, cytarabine, daunorubicin or idarubicin	Bleximenib ≥ 30 mg BID (oral) Cytarabine 200 mg/m^2^/day + Daunorubicin 60 mg/m^2^/day IV or Idarubicin 12 mg/m^2^/day IV (7 + 3 regimen) Consolidation: Intermediate-dose cytarabine + Bleximenib Maintenance: Bleximenib up to 12 months (if no transplant)	22 newly diagnosed AML patients 11 with NPM1 mutation, 11 with KMT2A rearrangement	ORR: 93% CR: 79%, CR/CRh: 86% ORR by genotype: - NPM1m: 100%, - KMT2Ar: 83%	- Febrile neutropenia: 64% -Thrombocytopenia: 68% - Anemia, neutropenia: 41% each - Leukopenia: 27%	Recruiting	NCT05453903
Issa GC et al., Blood 2024 [127]	Phase I/II, interventional, non-randomized, open-label, multi-center	Phase I/II Study of the ALL-Oral Combination of Revumenib (SNDX-5613) with Decitabine/Cedazuridine (ASTX727) and Venetoclax (SAVE) in R/R AML	Revumenib: 163 mg BID (oral, Days 1–28) Venetoclax: 400 mg daily (Days 1–28) Decitabine/Cedazuridine: 35 mg/100 mg daily	21 patients with relapsed/refractory AML Median age: 67 years (range 22–80) 76% with KMT2A rearrangement, 19% with NPM1 mutation 76% had prior venetoclax exposure 24% had prior menin inhibitor treatment	Overall Response Rate (ORR): 67% CR/CRh: 38% Median time to response: 1 cycle ORR in menin inhibitor–naïve patients: 75%	Febrile neutropenia: 29% Anemia: 24% Thrombocytopenia: 24% Neutropenia: 19%	Recruiting	NCT05360160
Issa GC et al., J Clin Oncol 2025 [118]	Phase I, II, open-label, interventional, dose- escalation, dose- expansion, sequential assignment	A Study of Revumenib in R/R Leukemias Including Those With an MLL/KMT2A Gene Rearrangement or NPM1 Mutation (AUGMENT-101)	Revumenib: 163 mg PO BID (oral, twice daily)	- Total patients treated at RP2D: 57 - Diagnosed with relapsed/refractory acute leukemia - 94.7% had acute lymphoblastic leukemia (ALL) - 89.5% had KMT2A rearrangement	CR or CRh: 17 patients (29.8%) Median duration of response: 9.1 months MRD-negative response 14/17 patients (82.4%)	- Differentiation syndrome: 14.0% (any grade), 7.0% grade 3 - QTc prolongation: 12.3% grade ≥3 - Febrile neutropenia: 21.1% - Anemia: 15.8% -Thrombocytopenia: 12.3% - Neutropenia: 12.3%	Recruiting	NCT04065399
Kretschmer L et al., Blood 2024 [128]	Phase II, randomized, interventional	Venetoclax plus Azacitidine vs. Intensive Chemotherapy for fit patients with newly diagnosed NPM1-mutated AML (VINCENT)	Venetoclax (VEN): 400 mg PO QD on days 1–28 (initial ramp-up) Azacitidine (AZA): 75 mg/m^2^ SC QD on days 1–7 SOC (Standard of Care) Arm: DA + GO: Cytarabine 200 mg/m^2^ (days 1–7), Daunorubicin 60 mg/m^2^ (days 3–5), Gemtuzumab Ozogamicin 3 mg/m^2^ (days 1, 4, 7) HAM (for non-responders): Cytarabine 1000–3000 mg/m^2^ BID (days 1–3), Mitoxantrone 10 mg/m^2^ (days 3–5) IDAC (consolidation): Cytarabine 1000–1500 mg/m^2^ BID (days 1–3)	Newly diagnosed NPM1-mutated AML, FLT3-wildtype Sample Size: 146 patients randomized 1:1 (VEN/AZA vs. SOC) Enrollment started: April 2024; currently 18 centers active	Not yet reported	Not yet reported	Recruiting	NCT05904106
Sartor C et al., Blood 2024 [129]	Phase II, non-randomized, interventional, open-label	Venetoclax and Azacitidine for the management of molecular relapse/progression in adult NPM1-mutated AML: VEN-AZA as a bridge to transplant therapy (single arm)	Venetoclax (VEN): 400 mg PO QD on days 1–28 Azacitidine (AZA): 75 mg/m^2^ SC or IV on days 1–7 of each cycle	Total screened: 24 patients Eligible/enrolled: 20 patients Analyzed: 15 patients All in molecular relapse (NPM1-mutated AML), confirmed complete remission Median prior intensive chemotherapy cycles: ≥2 ECOG PS = 0 33% had FLT3-ITD, 20% had FLT3-TKD mutations at diagnosis	MRD-negative (NPM1m < 0.01%): 9/15 (60%) Molecular response ≥1 log reduction: 3/15 (20%) Overall molecular response: 80% Morphologic relapse: 0% while on treatment Bridged to alloSCT in CR or better: 13/15 (87%) Median time to MRDneg: 1.64 months Median time to alloSCT: 3.45 months All patients alive and in CR at 9.8-month median follow-up	Hematologic AEs ≥ G3: 7/15 patients (47%) - Neutropenia: 10 events -Thrombocytopenia: 3 events - Pancytopenia: 1 event Febrile neutropenia G3: 1 patient (7%)	Unknown	NCT04867928

**Table 3:** Clinical trials in NPM1-mutated AML (Clinicaltrials.gov, accessed on 1 June 2025).

NCT Identifier	Current Status	Phase and Type of the Trial	Intervention
1. NCT04689815	Unknown	Prospective, open-label, phase II, interventional, single-group	Oral arsenic trioxide for NPM1-mutated AML: oral arsenic trioxide, ascorbic acid plus azacitidine (one arm)
2. NCT05020665	Terminated, due to challenges associated with study enrollment or with post-COVID impacts	Phase III, interventional, randomized, double-blind, placebo-controlled study	Entospletinib (SYK inhibitor) in combination with intensive induction and consolidation chemotherapy in adults with newly diagnosed NPM1-mutated AML: Intensive chemotherapy plus entospletinib vs. intensive chemotherapy plus placebo
3. NCT04867928	Unknown	Phase II, non-randomized, interventional, open-label	Venetoclax and Azacitidine for the management of molecular relapse/progression in adult NPM1-mutated AML: VEN-AZA as a bridge to transplant therapy (single arm)
4. NCT04931992	Unknown	Retrospective, observational	Outcome of patients with CBF and/or NPM1-mutated AML in first molecular relapse
5. NCT05904106	Recruiting	Phase II, randomized, interventional	Venetoclax plus Azacitidine vs. intensive chemotherapy for fit patients with newly diagnosed NPM1-mutated AML (VINCENT)
6. NCT01237808	Completed, no results posted	Phase III, randomized, two-arm, open-label, interventional, multicenter	Study of low-dose cytarabine and etoposide with or without all-trans retinoic acid in older patients not eligible for intensive chemotherapy with acute myeloid leukemia and NPM1 mutation: low-dose cytarabine-etoposide without ATRA vs. low-dose cytarabine-etoposide with ATRA
7. NCT05886049	Recruiting	Phase I, interventional	Testing the addition of an anti-cancer drug, SNDX-5613 to the standard chemotherapy treatment (daunorubicin and cytarabine) for newly diagnosed patients with acute myeloid leukemia that has changes in NPM1 or MLL/KMT2A gene
8. NCT00893399	Completed, no results posted	Phase III, randomized, two-arm, open-label, interventional	Study of chemotherapy in combination with all-trans retinoic acid (ATRA) with or without gemtuzumab ozogamicin in patients with acute myeloid leukemia (AML) and mutant nucleophosmin-1 (NPM1) gene mutation: chemotherapy with ATRA and GO vs. chemotherapy with ATRA without GO
9. NCT06001788	Recruiting	Interventional, phase I, non-randomized, open-label	Safety and tolerability of ziftomenib combinations in patients with relapsed/refractory acute myeloid leukemia: Ziftomenib plus FLAG-IDA, Ziftomenib plus low-dose cytarabine, Ziftomenib plus gilteritinib
10. NCT05738538	Available	Expanded access	Expanded access to ziftomenib
11. NCT01835288	Withdrawn	Phase II, interventional	Arsenic trioxide in treating patients with relapsed or refractory acute myeloid leukemia
12. NCT04988555	Recruiting	Phase I, II, open-label, interventional, dose-escalation, dose-expansion, non-randomized	A study of DSP-5336 in relapsed/refractory AML/ALL with or without MLL rearrangement or NPM1 mutation
13. NCT05453903	Recruiting	Phase Ib, interventional, non-randomized, 3 arms	A study of bleximenib (JNJ-75276617) in combination with acute myeloid leukemia (AML) directed therapies: Arm A (R/R): JNJ-75276617, VEN, AZA Arm B (ND, chemo-ineligible): JNJ-75276617, VEN, AZA Arm C (ND, chemo-eligible): JNJ-75276617, cytarabine, daunorubicin or idarubicin
14. NCT05735184	Recruiting	Phase I, non-randomized, interventional, open-label, parallel assignment	A study to investigate the safety and tolerability of Ziftomenib in combination with venetoclax/azacitidine, venetoclax, or 7 + 3 in patients with AML: Ziftomenib/VEN/AZA (R/R) Ziftomenib/7 + 3 (first line) Ziftomenib/VEN/AZA (first line) Ziftomenib/VEN (R/R)
15. NCT04065399	Recruiting	Phase I, II, open-label, interventional, dose-escalation, dose-expansion, sequential assignment	A study of Revumenib in R/R leukemias including those with an MLL/KMT2A gene rearrangement or NPM1 mutation (AUGMENT-101)
16. NCT05521087	Withdrawn	Phase I/Ib, interventional, non-randomized	A study of JNJ-75276617 in combination with conventional chemotherapy for pediatric and young adult participants with relapsed/refractory acute leukemias
17. NCT05153330	Active, not recruiting	Phase I, interventional, non-randomized	Study of BMF-219, a covalent menin inhibitor, in adult patients with AML, ALL (with KMT2A/MLL1r, NPM1 mutations), DLBCL, MM, and CLL/SLL
18. NCT05326516	Completed	Phase I, non-randomized, open-label, interventional, dose-escalation, parallel assignment	A study of Revumenib in combination with chemotherapy in participants with relapsed/refractory acute leukemia (AUGMENT-102)
19. NCT04067336	Recruiting	Phase I, II, randomized, open-label, parallel assignment, interventional, dose-escalation, dose-validation/expansion	First in human study of ziftomenib in relapsed or refractory acute myeloid leukemia (KO-MEN-001)
20. NCT05918692	Recruiting	Phase I, non-randomized, open-label, interventional, dose-escalation, dose-expansion, parallel assignment	A phase 1, study of BMF-500 in adults with acute leukemia
21. NCT06226571	Recruiting	Phase I, open-label, interventional, dose-escalation, dose-expansion	A study of SNDX-5613 (revumenib) in combination with intensive chemotherapy in participants with acute myeloid leukemias
22. NCT06313437	Recruiting	Phase I, non-randomized, open-label, interventional, single-arm	Revumenib in combination with 7 + 3 + midostaurin in AML
23. NCT06652438	Recruiting	Phase III, randomized, interventional, single-group	Revumenib in combination with azacitidine + venetoclax in patients NPM1-mutated or KMT2A-rearranged AML
24. NCT06930352	Not Yet Recruiting	Phase II, interventional, single-group	Ziftomenib for the treatment of patients with NPM1 mutated or KMT2A rearranged acute myeloid leukemia not eligible for standard therapy
25. NCT06440135	Recruiting	Phase I, open-label, interventional, single-group	Ziftomenib maintenance post allo-HCT
26. NCT06222580	Recruiting	Phase I, open-label, interventional	SNDX-5613 and gilteritinib for the treatment of relapsed or refractory FLT3-mutated acute myeloid leukemia and concurrent MLL-rearrangement or NPM1 mutation
27. NCT06268574	Recruiting	Phase II, open-label, interventional, single-group	Safety and efficacy of RVU120 for treatment of relapsed/refractory AML (RIVER-52)
28. NCT06424340	Recruiting	Phase I, II, open-label, interventional, single-group	MB-dNPM1-TCR.1 in relapsed/refractory AML
29. NCT06852222	Not Yet Recruiting	Phase III, randomized, double-blind, parallel assignment, interventional, placebo-controlled	A study of bleximenib, venetoclax and azacitidine for treatment of participants with acute myeloid leukemia (AML) (cAMeLot-2)
30. NCT06575296	Recruiting	Phase I, open-label, interventional, single-group	Revumenib for the treatment of acute leukemia in patients post-allogeneic stem cell transplant
31. NCT06652438	Recruiting	Phase III, randomized, interventional, single-group	Revumenib in combination With Azacitidine + Venetoclax in Patients NPM1-mutated or KMT2A-rearranged AML
32. NCT05886049	Recruiting	Phase I, open-label, single-group	Testing the addition of an anti-cancer drug, SNDX-5613, to the standard chemotherapy treatment (Daunorubicin and Cytarabine) for newly diagnosed patients with acute myeloid leukemia that has changes in NPM1 or MLL/KMT2A gene
33. NCT05918913	Available	Expanded access	Expanded access program for Revumenib
34. NCT06440135	Recruiting	Phase I, open-label, interventional, single-group	Ziftomenib maintenance post allo-HCT
35. NCT06376162	Recruiting	Phase I, open-label, interventional, single-group	Ziftomenib in combination with chemotherapy for children with relapsed/refractory acute leukemia

## Abbreviations

A	
AZA	Azacitidine
ALCL	Anaplastic large cell lymphoma
ALL	Acute Lymphoblastic Leukemia
AML	Acute Myeloid Leukemia
ANKRD26	Ankyring Repeat Domain Containing protein 26
APL	Acute promyelocytic leukemia
ARF	Alternative Reading Frame
ASXL1	Additional Sex Combs-like 1
ATO	Arsenic trioxide
ATRA	All-trans retinoic acid
B	
BCL-2	B-cell lymphoma-2
BCL-XL	B-cell lymphoma extra large
BCOR	BCL6 corepressor
BCORL1	BCL6 corepressor-like 1
BM	Bone Marrow
BRAF	V-Raf Murine Sarcoma Viral Oncogene Homolog B1
C	
CAR	Chimeric Antigen Receptor
CBF	Core Binding Factor
CBL	Casitas B-lineage Lymphoma
CD	Cluster of Differentiation
CEBPA	CCAAT enhancer binding protein alpha
CH	Clonal Hematopoiesis
CNS	Central Nervous System
CR	Complete Remission
CRISPR	Clustered Regularly Interspaced Short Palindromic Repeats
CSF3R	Colony Stimulating Factor 3 receptor
D	
DDX41	DEAD Box helicase 41
DNA	Deoxyribonucleic acid
DNMT3A	*DNA (cytosine-5)-methyltransferase 3A*
E	
EFS	Event-Free Survival
ELN	European Leukemia Net
ETV6	ETS variant transcription factor 6
EZH2	Enhancer of Zeste Homolog 2
F	
FAB	French–American–British Classification
FDA	Food and Drug Administration
FLT3-ITD	FMS-like tyrosine kinase 3 internal tandem duplication
FLT3-TKD	FMS-like tyrosine kinase 3 tyrosine kinase domain
FOXM1	Forkhead box M1
G	
GATA2	GATA Binding protein 2
GPR56	G protein coupled receptor 56
GO	Gemtuzumab ozogamicin
H	
HDAC	Histone Deacetylase
HiDAC	High-Dose Cytarabine
HLA	Human Leukocyte Antigen
HLF	Hepatic Leukemia Factor
HMA	Hypomethylating agent
HSCT	Hematopoietic Stem Cell Transplantation
HOX	Homeobox
I	
ICC	International Consensus Classification
IDAC	Intermediate Dose Cytarabine
IDH1	Isocitrate Dehydrogenase 1
IDH 2	Isocitrate Dehydrogenase 2
IHC	Immunohistochemistry
J	
JAK2	Janus Kinase 2
K	
KMT2A	Lysine Methyltransferase 2A
KIT	KIT protooncogene, receptor tyrosine kinase
KPT-330	Selinexor
KPT-8602	Eltaxenor
KO	Knockout
KO-539	Ziftomenib
KRAS	Kirsten rat sarcoma viral oncogene homolog
M	
MDS	Myelodysplastic Syndrome
MCL1	Myeloid Cell Leukemia sequence 1
MEIS1	Myeloid Ecotropic viral Integration site 1
MEIS-SMC4	Myeloid Ecotropic viral Integration site 1-Structural Maintenance of Chromosome 4
MLL	Mixed Lineage Leukemia
MPNs	Myeloproliferative Neoplasms
MRGs	Myelodysplasia related gene mutations
MRD	Measurable Residual Disease
MRD-LL	Measurable Residual Disease at Low Level
N	
NCCN	National Comprehensive Cancer Network
ND	Newly Diagnosed
NF1	Neurofibromatosis type 1
NGS	Next-Generation Sequencing
NHL	Non-Hodgkin lymphoma
NK	Natural Killer
NPM1	Nucleophosmin 1
NPM1wt	NPM1 wild type
NRAS	Neuroblastoma RAS viral oncogene homolog
O	
ORR	Overall Response Rate
OS	Overall Survival
P	
PB	Peripheral Blood
PBX3	Pre-B-cell leukemia transcription factor 3
PD-1	Programmed (Cell) Death 1
PD-L1	Programmed (Cell) Death Ligand 1
PFS	Progression-free survival
PHF6	Plant Homeodomain Finger Protein 6
PPM1D	Protein phosphatase magnesium-dependent 1 delta
PRC1	Polycomb repressive complex 1
PRC2	Polycomb repressive complex 2
PTPN11	Protein Tyrosine Phosphatase Non-Receptor Type 11
R	
RNA	Ribonucleic acid
RP2D	Recommended phase 2 dose
R/R AML	Relapsed or refractory acute myeloid leukemia
RT-qPCR	Quantitative Reverse Transcription Polymerase Chain Reaction
RUNX1	Runt-related transcription factor 1
S	
SETBP1	Set Binding Protein 1
SF3B1	Splicing Factor 3B Subunit 1
SINE	Selective Inhibitors of Nuclear Export
SNDX-5613	Revumenib
SRSF2	Serine/Arginine-rich Splicing Factor 2
STAG2	Stromal Antigen 2
STAT5	Signal transducer and activator of transcription 5
T	
TCR	T-cell receptor
TET2	TET methylcytosine dioxygenase 2
TP53	Tumor Protein p53
U	
U2AF1	U2 small nuclear RNA auxiliary factor 1
V	
VAF	Varied Allele Frequency
VEN	Venetoclax
W	
WHO	World Health Organization
WT1	Wilms Tumor 1
X	
XPO1	Exportin 1
Z	
ZRSR2	Zinc finger (CCCH type), RNA-binding motif and serine/arginine rich 2
